# Perceptions of stimulant and buprenorphine diversion and strategies to address it

**DOI:** 10.1093/haschl/qxae074

**Published:** 2024-06-03

**Authors:** Lori Uscher-Pines, Jessica Sousa, Pushpa Raja, Ateev Mehrotra, Alisa B Busch, Haiden A Huskamp

**Affiliations:** RAND Corporation, Arlington, VA, 22202, United States; RAND Corporation, Boston, MA, 02116, United States; Department of Mental Health, Greater Los Angeles VA Medical Center, Los Angeles, CA 90073, United States; Department of Health Care Policy, Harvard Medical School, Boston, MA 02115, United States; Internal Medicine, Beth Israel Deaconess Medical Center, Boston, MA 02215, United States; Department of Health Care Policy, Harvard Medical School, Boston, MA 02115, United States; Department- Psychiatry, McLean Hospital, Belmont, MA 02478, United States; Department of Health Care Policy, Harvard Medical School, Boston, MA 02115, United States

**Keywords:** telemedicine, telehealth, buprenorphine, stimulants, controlled substances, diversion

## Abstract

There is ongoing policy debate on the prescribing of controlled substances such as buprenorphine and stimulants via telemedicine. The goal of federal and state policymakers is to ensure access to care while limiting diversion risk. However, there is little evidence on how clinicians view and address diversion and on telemedicine's role in diversion. From December 2023 to January 2024, we conducted semi-structured interviews with 21 psychiatrists and primary care physicians engaged in hybrid (telemedicine and in-person) care models in which we explored perceptions of diversion and strategies used to monitor for diversion. Most physicians reported monitoring for diversion, but there was little consistency on how monitoring was done and reported strategies did not differ between telemedicine vs in-person care. When physicians suspected diversion, there was also wide variation in responses: some clinicians did not immediately take any action while others imposed more requirements on patients (e.g., more frequent visits), no longer prescribed the controlled substance, or terminated the patient from their practice. Few physicians had ever reported a case of suspected diversion to law enforcement. Our findings suggest that the Drug Enforcement Administration could clarify reporting requirements and professional societies could provide additional guidance on how to respond to suspected diversion, given the current variation in practice across clinicians could be exploited by individuals who want to divert.

## Introduction

The US Drug Enforcement Administration (DEA) has a mission to prevent, detect, and investigate diversion of controlled medications while ensuring an adequate supply for legitimate medical needs.^1^ This mission has become especially challenging in the context of the opioid epidemic and increasing overdose deaths. Diversion is defined as the unauthorized rerouting or misappropriation of prescribed controlled medications to someone other than the person for whom it was intended. Examples of diversion include giving a prescribed medication to a friend or family member or selling the medication illicitly. Diversion is common in the United States. A 2008 systematic review found that 16%-29% of individuals with stimulant prescriptions were asked to give, sell, or trade their medications at some point in their lifetime.^2^ Guidance from the Department of Health and Human Services and law enforcement agencies include language that clinicians “should report” suspected diversion to law enforcement.^3,4^ In cases of significant loss or theft of controlled substances, reporting is mandatory.^5^

Although there is little empirical evidence on the issue, some policymakers are concerned that the dramatic shift to telemedicine for behavioral health care that occurred with the start of the COVID-19 pandemic may increase diversion risk. Prior to the pandemic, the 2008 Ryan Haight Act dictated that clinicians could not prescribe controlled medications via telemedicine without first conducting an in-person examination. During the pandemic, this restriction was temporarily waived, facilitating the growth of prescribing of controlled substances via telemedicine and telemedicine-only care models and companies.^6-9^ The pandemic-era flexibilities are currently set to expire in December 2024 unless the DEA issues new rules or implements another extension.

The DEA and many states are now actively debating whether to revert to prepandemic requirements for an in-person visit or to permanently eliminate this requirement. If the requirement is eliminated on a permanent basis, there is also the question of whether new guardrails should be introduced to reduce diversion risk. A key challenge is the paucity of research on the impact of telemedicine on diversion, the effectiveness of a required in-person visit in reducing diversion, and how clinicians think about diversion and the strategies they implement to address it for telemedicine vs in-person visits. Only a handful of surveys have explored clinician beliefs and strategies on diversion, and they all predate the large-scale shift to telemedicine.^10,11^ To help fill this knowledge gap, we assessed how physicians prescribing stimulants and buprenorphine monitor for and address diversion, how this varies for telemedicine and in-person visits, and their views on policy approaches to maintaining access to care while simultaneously minimizing diversion risk.

## Data and methods

We conducted purposive, criterion sampling using WebMD's national physician panel. The panel is frequently used in federally funded research studies and includes more than 700 000 US-based physicians who join the panel to access clinical content and continuing medical education activities.^12^ To be eligible, a participant had to be a primary care physician (PCP) or psychiatrist engaged in hybrid care models (some telemedicine and some in-person care) in outpatient settings. They also had to prescribe stimulants and/or buprenorphine for the treatment of opioid use disorder to at least 4 adult patients in the prior year. Physicians employed by the Indian Health Service, Veterans Affairs, and Military Health System were excluded. We focused on stimulants (Schedule II medications) and buprenorphine (Schedule III) for this study because they have been the focus of recent policy discussions. The DEA investigated the inappropriate prescribing practices of stimulants by several telemedicine companies starting in 2022.^13^ Further, it issued a proposed rule specifically for buprenorphine in 2023.^14^

In November 2023, WebMD emailed information about the study opportunity to 600 PCPs and psychiatrists who had opted to participate in research. Sixty completed an 8-question eligibility survey, and 23 were found eligible. We continued to schedule and conduct interviews until we reached thematic saturation, the point at which additional interviews did not reveal new themes.

Eligible physicians were invited to participate in a 60-min semi-structured interview and received a $200 Amazon gift card. Interviews were conducted via Microsoft Teams and occurred from December 2023 to January 2024. The interview protocol covered perceptions about diversion and the role of telemedicine in diversion, strategies to monitor for and prevent diversion, experiences reporting cases of suspected diversion to law enforcement, and policy recommendations on telemedicine for prescribing controlled substances. Participants were asked to comment on their views and practices when prescribing buprenorphine for the treatment of opioid use disorder and stimulants for the treatment of attention deficit hyperactivity disorder (ADHD). Further, because clinic level, state, and/or federal policy can influence prescribing practices, we asked participants to clarify extent to which they were able to set policy themselves vs were subject to external requirements. Interviews were recorded and transcribed. Participants provided verbal informed consent.

We analyzed interview data by using inductive thematic analysis.^15^ We first conducted open coding of interview transcripts, followed by axial coding to establish connections among themes. The lead author (L.U.-P.) developed the initial codebook by reviewing 8 transcripts. The codebook was then discussed, refined, and finalized in group meetings among 2 members of the study team (L.U.-P., J.S.). The lead author then coded all transcripts using Dedoose data analysis software. Following initial coding, we conducted matrix analysis in which rows listed participants and columns listed codes. Such matrices have been used in qualitative data analysis to efficiently identify similarities, differences, and trends in responses across groups of informants.^16^ A matrix provides a visual data display that enables the search for and a detailed analysis of patterns, themes, and other relationships and informs conclusions.^17^ Here, the matrix allowed us to interpret each participant's comments in the context of the type of medications they were prescribing (stimulants, buprenorphine, both). We identified themes through well-established techniques, including repetition (eg, if a concept was expressed more than 3 times) and emphasis (eg, if participants particularly engaged with or dedicated significant time to a concept). It should be noted that because multiple themes applied to both buprenorphine and stimulants (eg, barriers to reporting suspected diversion to law enforcement), we chose not to differentiate between the 2 medications in all themes. In cases where we observed differences by medication type, we clarified those differences in the Results section.

The research team included 6 health services researchers, including 2 psychiatrists, 1 internal medicine physician, 1 licensed clinical social worker, and 1 health economist, who have studied the role of telemedicine in access, quality, and costs for over a decade. Members of the research team have also submitted public comments to the DEA and participated in DEA listening sessions to summarize their prior work on telemedicine for the treatment of opioid use disorder. L.U.-P. and J.S. reflected on how their subjectivity and context influenced the research at multiple points in the process. They entered this work with the belief that policymakers have the responsibility to increase access to care through telemedicine and to prevent large-scale diversion. Further, although both goals are of critical importance and can be at odds, some stakeholders implicitly prioritize 1 goal over the other. The team had collaborative discussions that probed personal perspectives on the role of regulation in the practice of medicine, and how much regulation should curb clinician autonomy in the context of the opioid epidemic.

### Limitations

This study has several limitations. First, by design, all clinicians in the sample were engaged in hybrid care models with adult patients, and their views may differ from those who have shifted to telemedicine-only models. Research has shown that approximately 13% of all mental health specialists delivered telemedicine-only care in 2022.^18^ Clinicians who have shifted to telemedicine-only may be more comfortable initiating medication treatment without an in-person visit and may not see telemedicine as posing a greater diversion risk. Second, we did not capture the views of other prescribers of buprenorphine and stimulants including pediatricians, nurse practitioners, and physicians’ assistants. Third, responses may be influenced by social desirability bias. Even though we told participants that their responses were confidential, and we are not evaluating them, they might have given some answers that they perceived as “correct” vs accurate descriptions of their beliefs and practices. Fourth, we did not collect data on the number of patients in a participant's panel who received buprenorphine and/or stimulants each year. It is likely that views and practices may differ for lower vs higher volume prescribers.

## Results

Twenty-one physicians (10 psychiatrists and 11 PCPs) from 15 different states participated in an interview. Fifteen (71%) participants prescribed both stimulants and buprenorphine, and 6 (19%) prescribed stimulants only. No participants prescribed buprenorphine only. Two-thirds (*n* = 14, 66%) delivered 50% or fewer of their total visits via telemedicine (Table 1). Themes are presented below and summarized in Table 2.

**Table 1.** Participant characteristics (*N* = 21).

	*N*	%
Physician specialty		
Psychiatrist	10	48%
Internal medicine	4	19%
Family medicine	7	33%
Region		
West	6	29%
South	5	24%
Northeast	4	19%
Midwest	5	24%
% Telemedicine visits		
6%-50%	14	67%
51%-89%	5	24%
90%-95%	2	10%
Practice setting		
Solo private practice	5	24%
Nonhospital-based group private practice	7	33%
Hospital-based outpatient clinic	5	24%
Community health center	3	14%
Other outpatient setting	1	5%
Medications prescribed		
Both stimulants and buprenorphine	15	71%
Buprenorphine only	0	0%
Stimulants only	6	29%

**Table 2.** Summary of themes.

Theme 1	Many physicians in the sample felt diversion was common and problematic and that they had some responsibility to address it
Theme 2	Many physicians in the sample believed that telemedicine increased the potential for diversion; however, participants generally applied the same strategies to address diversion for both in-person and telemedicine visits
Theme 3	Physicians in the sample reported monitoring patients for diversion, but specific strategies varied
Theme 4	Many did not initiate stimulants or buprenorphine via telemedicine, but not necessarily because of diversion concerns
Theme 5	When physicians suspected diversion, there was wide variation in responses, including in actions they would take, how quickly they would take them, and in what order
Theme 6	Very few physicians had ever reported a case of suspected diversion to law enforcement and most identified numerous barriers to reporting
Theme 7	Some supported in-person visit requirements while others advocated for permeant flexibility to prescribe via telemedicine with the introduction of certain guardrails

*Many physicians in the sample felt diversion was common and problematic and that they had some responsibility to address it.* Multiple participants believed that diversion of buprenorphine and stimulants occurred frequently, and that stimulants diversion was particularly common among college students. As explained by a PCP in Maine, “You always hear patients tell you, ‘Oh, I got it [stimulants] from a friend.’” Nonetheless, a handful of physicians did not feel that diversion was common in their patient population and provided various rationales. For example, some described serving higher-income patients who did not seem to have financial motivation to sell medications. Some reported knowing their patients well and trusting them not to divert or felt that stimulant shortages motivated patients to hold on to their supply.

While many physicians in the sample felt that stimulant diversion was a societal problem that should be addressed, there was less consensus on whether buprenorphine diversion was a problem. A psychiatrist from Arkansas explained, “Buprenorphine lowers the chance that a patient has an overdose; whether it is prescribed, or they are buying it on the street.” Physicians also reported that misuse [not taking medication as prescribed] was more clinically important and more within their lane of responsibility than diversion. A psychiatrist from New York explained, “It is more important for me to impact misuse [because it is clearly a clinical issue] vs. diverting to someone else.”

Multiple participants in the sample felt that prescribing physicians bear some, but not all, responsibility for addressing diversion; they felt they share responsibility with others in the system including pharmacists and law enforcement. Also, given difficulties in detecting diversion, distinguishing it from misuse, and intervening to influence patient behavior, they felt they could not be held entirely responsible. A psychiatrist from North Carolina remarked, “We need to do due diligence, but… we can’t control what people do once they leave our office.”

Few physicians reported receiving any formal, didactic training on diversion, and several reported a lack of official policies or guidelines to inform practice-level monitoring and mitigation strategies.

*Many physicians in the sample believed that telemedicine increased the potential for diversion; however, participants generally applied the same strategies to address diversion for both in-person and telemedicine visits.* Many physicians argued that telemedicine increased diversion risk; however, this belief was not universal. Physicians believed that with telemedicine (which can include videoconferencing or audio-only visits), it can be easier for patients misrepresent themselves and to “doctor shop” (ie, see multiple prescribers for the same type of medication). Further, clinicians have fewer tools to do reliable urine drug screening. Several remarked that telemedicine is more transactional and the relationship with the physician is less personal, which they felt increases both the likelihood that a patient will divert and the likelihood that they will respond truthfully when confronted about suspected diversion. In addition, physicians reported that with telemedicine, a family member who is diverting stimulants from the patient can be off-camera during the visit and influencing the interaction in a way that is not discernible to the clinician. Finally, multiple physicians expressed particular concern about telemedicine-only companies that are motivated by profit. They explained that telemedicine-only care is often associated with fewer requirements (eg, less urine drug screening, fewer visits), so patients who seek to divert small quantities face fewer obstacles. A PCP from Maine said, “[Patients who leave us and go to telemedicine-only companies], I’m not saying those people will divert… I’m just saying we are lowering the barrier for them to divert.”

However, some felt that telemedicine was just another “access point” without any greater inherent diversion risk, and the same prevention strategies could be applied to both telemedicine and in-person care. A psychiatrist from Maryland explained, “So I get the DEA saying they want to put some limits on this [telemedicine], but I don't think there's any greater likelihood of diversion occurring via telehealth; and diversion was occurring long before telehealth ever became popularized… Patients can lie to us in person if they want.” A few physicians even pointed out the distinct advantages of telemedicine for reducing diversion risk. For example, the same psychiatrist also noted that telemedicine can make it safer for physicians to have difficult conversations because, you “don’t need to worry the patient will get aggressive” when confronted about suspected diversion.

Multiple physicians in the sample reported applying the same diversion monitoring strategies to both in-person and telemedicine visits. Only 1 participant, a PCP from California, reported conducting fewer urine drug screens with patients receiving buprenorphine via telemedicine than with in-person care.

*Physicians in the sample reported monitoring patients for diversion, but specific strategies varied.* Many physicians in the sample reported actively monitoring for diversion, and typically mentioned 1 or 2 strategies that they used. Checking the state's prescription drug monitoring program (PDMP) for evidence of receiving controlled medications from multiple prescribers was the most common. Beyond that, there was little consistency. Participants generally said that the strategies they implemented were a personal decision and that they used their own judgment in selecting monitoring strategies; however, a handful of physicians mentioned that the use of behavior contracts and the frequency with which they checked the PDMP were dictated by state law. Table 3 shows the full list of strategies used by 1 or more participants.

**Table 3.** Strategies to monitor for or address diversion.^a^

Strategies for stimulants	Require neuropsychological evaluations
	Meet with patient without family members (eg, to ask questions about a family member's role in preventing diversion)
Strategies for buprenorphine	Conduct urine drug screens (ie, to detect buprenorphine and metabolites)
	Require patient to file a police report if they report that their medication was stolen
	Only prescribe buprenorphine/naloxone (no buprenorphine monoproduct)
	Require patients to use a particular pharmacy with approved policies (eg, no early refills)
	Do Internet searches on patients to obtain information about criminal history related to diversion (eg, in cases of suspected drug distribution or manufacturing)
Strategies for both stimulants and buprenorphine	Check prescription drug monitoring program
	Require patient to sign a behavior contract
	Require in-person visits (initially and/or at some regular interval)
	Use advanced fill function
	Require pill counts
	Prescribe a smaller supply (eg, 2 vs 4 weeks)
	Prescribe the lowest effective dose

^a^This table shows the full list of strategies that were mentioned by one or more participants.

Only a handful of physicians did *not* proactively monitor all patients for diversion and instead were more reactive, taking action only when faced with clear evidence. Reasons for a lack of proactive monitoring included knowing patients well (and trusting them not to divert) and “not looking for problems.” Several stated that by assessing diversion risk at the first (typically in-person) visit, they could “weed out” patients they felt were more likely to divert and thus, not have to engage in ongoing monitoring. A psychiatrist from New York said, “I think a huge part of monitoring goes into my initial decision to prescribe or not.”

*Many did not initiate stimulants or buprenorphine via telemedicine, but not necessarily because of diversion concerns.* Multiple physicians in the sample required an in-person visit before they would prescribe buprenorphine or stimulants via telemedicine. Several reported that this was a clinic-level policy that all clinicians in their practice followed. Physicians had a variety of reasons for preferring to see patients in-person initially, and these were not limited to diversion concerns. Many discussed the benefits of an initial in-person visit for clinical care quality or accountability. A PCP from New Jersey explained, “It is very important to have the patient come in for that first visit. What if the patient is hypertensive and something happens to them- side effects wise. Having that [in-person] visit is important to go over the details [of starting a stimulant].”

*When physicians suspected diversion, there was wide variation in responses, including in actions they would take, how quickly they would take them, and in what order.* Physicians discussed 4 general responses, including allowing 1-3 “strikes” (eg, in cases of suspected deception, relapse, or diversion) before taking action, using a “shorter leash” (eg, requiring more frequent urine drug screens or visits), discontinuing prescribing the diverted medication, and terminating the patient from the practice. Of note, some were willing to terminate patients after other attempts at remediation, while others were not. A PCP from Wisconsin said, “I don’t fire a patient. I just say, ‘you won’t get stimulants anymore.’ They often then fire themselves.”

*Very few physicians had ever reported a case of suspected diversion to law enforcement and most identified numerous barriers to reporting.* Many physicians in the sample had never reported a case of suspected diversion to local law enforcement or DEA. Each participant shared 1-2 reasons why they had not chosen to report. Physicians listed more than 15 different reasons in total, suggesting numerous barriers to reporting (Table 4). One of the most common challenges that physicians mentioned was that they rarely had proof (only suspicion) that diversion had occurred.

**Table 4.** Reasons for not reporting cases of suspected diversion to law enforcement.

Views of law enforcement	Perception that police will not do anything if physician files a report; there will not be any follow through
	Lack of trust in the DEA and belief that they will treat this punitively, “like a crime”
Reporting process	Perception that it is time-consuming to report
	Belief that it is difficult to detect diversion and distinguish diversion from misuse; information that the physician has is often based on hearsay
	Claim that it is not clear when exactly to report/how much evidence is needed to justify reporting
Risks of reporting	Concerns about HIPAA or patient confidentiality
	Concern that if physician reports, they will get a reputation and lose patients
	Concern that if physician reports, the patient will take them to court for medical malpractice/sue for patient abandonment
Philosophical reasons	Belief that diversion of buprenorphine can be part of a recovery process; understanding that recovery is difficult
	Belief that it is not the role of the physician to report
	Belief that buprenorphine diversion is not dangerous
	View that clinicians deal with reports of illegal activities on a regular basis; diversion is not particularly unique
	View that it is futile to report because diversion will always exist
	Belief that diversion is not an ongoing problem: if the physician stops prescribing, that is the end of it
Other miscellaneous reasons	“I don’t even think about reporting it to law enforcement”
	“I am not even sure I am supposed to report”
	“I haven’t had any cases of diversion [more likely misuse]”

This table shows the full list of reasons that were mentioned by one or more participants.

*Some supported in-person visit requirements while others advocated for permeant flexibility to prescribe via telemedicine with the introduction of certain guardrails.* Multiple physicians felt that requiring an initial in-person visit was good policy and supported a return to the prepandemic, Ryan Haight Act requirement. Some felt that telemedicine-only care leads to a “lax approach to health” or “reduces respect for medication and its side effects.” Others felt that the in-person requirement would have the positive effect of limiting telemedicine companies. However, others disagreed and pointed to the importance of telemedicine for increasing access. A PCP from Michigan explained, “I would never initiate these drugs via telemedicine, but I think providers need to use their own judgement. There will always be exceptions—for example, patients without access to care in rural areas—when telemedicine is needed.”

If the DEA decides to allow telemedicine prescribing without an in-person visit, participants had various suggestions for guardrails that could be introduced to reduce diversion risk. However, for almost every guardrail endorsed by 1 participant, another would explain why that guardrail could be burdensome or ineffective. Examples of recommended guardrails included requiring: urine drug screening, behavior contracts, hybrid care models (eg, some in-person care), frequent checks of the PDMP, frequent visits, and special registration for prescribing clinicians that would allow them to bypass in-person visit requirements if registered with the DEA.^19^ Few physicians were knowledgeable about what special registration would entail. Some thought it was a reasonable approach to reduce diversion or that it was a fair compromise, whereas others thought it would be ineffective or overly burdensome and thus restrict access. According to a psychiatrist from Utah, “I don’t love it [special registration]. I don’t hate it. The limitations that they've had in the past on buprenorphine have been more restrictive than that. And so, if we end up in a place that is overall less restrictive than where we used to be, I think that that's probably better.”

## Discussion

Multiple physicians in this study monitored for diversion. They appreciated telemedicine's role in increasing access but acknowledged that telemedicine may lead to greater diversion. Specific monitoring strategies varied across physicians and when physicians suspected diversion, there was wide variation in responses. Physicians identified numerous barriers to reporting suspected diversion to law enforcement.

This study is the first that we are aware of to explore physicians’ perceptions and strategies to address stimulant and buprenorphine diversion since the widespread use of telemedicine. Prior surveys also documented variation in practices to address diversion and documented that some prescribers do not believe diversion is common or of significant concern.^10,11^ Colaneri et al^11^ showed that the leading diversion prevention strategies among stimulant prescribers included prescribing long-acting stimulants and nonstimulants. Lin et al^10^ showed that the leading practices among buprenorphine prescribers included urine drug screens, pill counts, and frequent visits.

Our findings have several policy implications for diversion prevention strategies in general. First, there should be more clarity on when clinicians must report suspected diversion to law enforcement. Current guidance states that clinicians “should report,”^3,4^ but lacks detail on when reporting small-scale diversion is in fact appropriate and which types of evidence justify the filing of a report. Given HIPAA and other privacy protections,^20^ it is not clear that physicians who proactively report (ie, absent a warrant or subpoena and an active investigation) would avoid sanction, particularly if they lacked sufficient evidence to prove diversion had occurred. Second, our results show that physicians are uncomfortable interfacing with law enforcement. It follows that improving tools that support clinicians in detecting and managing diversion among themselves may be the most fruitful approach. For example, steps could be taken to improve PDMPs (eg, implement alerts for potential “doctor shopping,” allow clinicians to access relevant data from the PDMPs of neighboring states, embed PDMPs within the EHR so that workflow is streamlined). Third, given wide variation in physicians’ responses to suspected diversion, states and professional organizations could provide more guidance to clinicians on when to stop prescribing, when to consider termination, and steps to take in confronting patients about suspected diversion. Current variability across clinicians is in itself problematic and can be exploited by individuals who want to divert.

Our study also emphasizes the quandary before the DEA in terms of how to regulate the prescribing of controlled substances via telemedicine. Most physicians in our study acknowledged that telemedicine could increase diversion, but some argued that re-instating the in-person visit requirement that was in place prior to the pandemic would reduce access to care. Access to care is a critical issue, with only 1 in 5 patients with opioid use disorder receiving medication treatment each year, and 10% of the U.S. population living more than 10 miles from the nearest buprenorphine prescriber.^21,22^

The DEA is also limited in what it can implement because unlike states, it does not have authority to regulate the practice of medicine. While there was variable enthusiasm for a special registration process among physicians in our sample, some considered it to be a good compromise. Given that establishing such a process is within the DEA's authority and there is little evidence on the implications of different policy approaches, setting up and subsequently evaluating the impact of a special registration process may be a reasonable approach. Future research should assess if a special registration process can reduce diversion without preventing prescribers from offering telemedicine and explore the role of telemedicine in diversion and the effectiveness of in-person visits in reducing diversion.

## Supplementary Material

qxae074_Supplementary_Data

## References

[1] U.S. Drug Enforcement Administration . About Us: Diversion Control Division; 2022. Accessed February 18, 2024. https://www.deadiversion.usdoj.gov/about-us.html

[2] Wilens TE , Adler LA, Adams J, et al Misuse and diversion of stimulants prescribed for ADHD: a systematic review of the literature. J Am Acad Child Adolesc Psychiatry. 2008;47(1):21–31. 18174822 10.1097/chi.0b013e31815a56f1

[3] U.S. Centers for Medicare & Medicaid Services . Drug Diversion Toolkit: What Is a Prescriber's Role in Preventing the Diversion of Prescription Drugs? Centers for Medicare & Medicaid Services; 2016.

[4] U.S. Department of Health and Human Services . Resource Guide: Prescription Drug Diversion. Department of Health and Human Services; 2016.

[5] U.S. Drug Enforcement Administration . Reporting theft or significant loss of controlled substances, final rule. Fed Regist. 2023;88(119):40707–40713. https://www.govinfo.gov/content/pkg/FR-2023-06-22/pdf/2023-13085.pdf

[6] Jones CM , Shoff C, Blanco C, Losby JL, Ling SM, Compton WM. Association of receipt of opioid use disorder-related telehealth services and medications for opioid use disorder with fatal drug overdoses among Medicare beneficiaries before and during the COVID-19 pandemic. JAMA Psychiatry. 2023;80(5):508–514. 36988913 10.1001/jamapsychiatry.2023.0310 PMC10061313

[7] Nguyen TD , Gupta S, Ziedan E, et al Assessment of filled buprenorphine prescriptions for opioid use disorder during the coronavirus disease 2019 pandemic. JAMA Intern Med. 2021;181(4):562–565. 33346795 10.1001/jamainternmed.2020.7497 PMC7754073

[8] Huskamp HA , Uscher-Pines L, Raja P, Normand ST, Mehrotra A, Busch AB. Trends in use of telemedicine for stimulant initiation among children and adults. Psychiatr Serv. 2024;appips20230421. doi: 10.1176/appi.ps.20230421. Epub ahead of print. PMID: 38239181. PMC11216869 38239181

[9] Danielson ML , Bohm MK, Newsome K, et al Trends in stimulant prescription fills among commercially insured children and adults—United States, 2016–2021. MMWR Morb Mortal Wkly Rep. 2023;72(13):327–332. 36995976 10.15585/mmwr.mm7213a1 PMC10078845

[10] Lin LA , Lofwall MR, Walsh SL, Gordon AJ, Knudsen HK. Perceptions and practices addressing diversion among US buprenorphine prescribers. Drug Alcohol Depend. 2018;186:147–153. 29573649 10.1016/j.drugalcdep.2018.01.015 PMC5911230

[11] Colaneri N , Keim S, Adesman A. Physician practices to prevent ADHD stimulant diversion and misuse. J Subst Abuse Treat. 2017;74:26–34. 28132697 10.1016/j.jsat.2016.12.003

[12] WebMD Medscape . Market research; 2021. Accessed June 3, 2024. https://www.medscape.com/sites/public/marketresearch

[13] Vaidya A. Report: Telehealth Company's prescribing practices come under DEA scrutiny. mHealth Intelligence. Accessed September 16, 2022. https://mhealthintelligence.com/news/report-telehealth-company-dones-prescribing-practices-come-under-dea-scrutiny

[14] U.S. Drug Enforcement Administration . Expansion of induction of buprenorphine via telemedicine encounter (proposed rule). Fed Regist. 2023;88(40):12890–12906. https://www.federalregister.gov/d/2023-04217

[15] Braun V , Clarke V. Using thematic analysis in psychology. Qual Res Psychol. 2006;3(2):77–101.

[16] Averill JB . Matrix analysis as a complementary analytic strategy in qualitative inquiry. Qual Health Res. 2002;12(6):855–866. 12109729 10.1177/104973230201200611

[17] Lambert-Kerzner A , Maynard C, McCreight M, et al Assessment of barriers and facilitators in the implementation of appropriate use criteria for elective percutaneous coronary interventions: a qualitative study. BMC Cardiovasc Disord. 2018;18(1):164. 30103677 10.1186/s12872-018-0901-6 PMC6205154

[18] Hailu R , Huskamp HA, Busch AB, Uscher-Pines L, Raja P, Mehrotra A. Characteristics of mental health specialists who shifted their practice entirely to telemedicine. JAMA Health Forum. 2024;5(1):e234982. 38277172 10.1001/jamahealthforum.2023.4982 PMC10818220

[19] Uscher-Pines L , Huskamp HA, Mehrotra A. Time for the DEA to implement a special registration process for telemedicine prescribing of buprenorphine. Health Affairs Forefront. 2023. 10.1377/forefront.20230831.931397

[20] U.S. Department of Health and Human Services . *HIPAA Administrative Simplification: Regulation Text*, 45 CFR Parts 160, 162, and 164. March 26, 2013. Accessed February 18, 2024. https://www.hhs.gov/sites/default/files/ocr/privacy/hipaa/administrative/combined/hipaa-simplification-201303.pdf

[21] Langabeer JR , Stotts AL, Cortez A, Tortolero G, Champagne-Langabeer T. Geographic proximity to buprenorphine treatment providers in the US. Drug Alcohol Depend. 2020;213:108131. 32599495 10.1016/j.drugalcdep.2020.108131

[22] Substance Abuse and Mental Health Services Administration . Key Substance Use and Mental Health Indicators in the United States: Results from the 2021 National Survey on Drug Use and Health. Center for Behavioral Health Statistics and Quality, Substance Abuse and Mental Health Services Administration; 2022 (HHS Publication No. PEP22-07-01-005, NSDUH Series H-57).

